# A booster of Delta-Omicron RBD-dimer protein subunit vaccine augments sera neutralization of Omicron sub-variants BA.1/BA.2/BA.2.12.1/BA.4/BA.5

**DOI:** 10.1080/22221751.2023.2179357

**Published:** 2023-02-27

**Authors:** Minrun Duan, Huixin Duan, Yaling An, Tianyi Zheng, Shengfeng Wan, Hui Wang, Xin Zhao, Lianpan Dai, Kun Xu, George F. Gao

**Affiliations:** a School of Life Sciences, Yunnan University, Kunming, People’s Republic of China; b Savaid Medical School, University of Chinese Academy of Sciences, Beijing, People’s Republic of China; c Zhejiang University School of Medicine, Hangzhou, People’s Republic of China; d CAS Key Laboratory of Pathogen Microbiology and Immunology, Institute of Microbiology, Chinese Academy of Sciences, Beijing, People’s Republic of China; e Beijing Institute of Biological Products Company Limited, Beijing, People’s Republic of China; f Research Network of Immunity and Health (RNIH), Beijing Institutes of Life Science, Chinese Academy of Sciences, Beijing, People’s Republic of China

**Keywords:** SARS-CoV-2, Omicron, ZF2001, RBD-dimer, vaccine

## Abstract

The SARS-CoV-2 Omicron variants of concern (VOCs) showed severe resistance to the early-approved COVID-19 vaccines-induced immune responses. The breakthrough infections by the Omicron VOCs are currently the major challenge for pandemic control. Therefore, booster vaccination is crucial to enhance immune responses and protective efficacy. Previously, we developed a protein subunit COVID-19 vaccine ZF2001, based on the immunogen of receptor-binding domain (RBD) homodimer, which was approved in China and other countries. To adapt SARS-CoV-2 variants, we further developed chimeric Delta-Omicron BA.1 RBD-dimer immunogen which induced broad immune responses against SARS-CoV-2 variants. In this study, we tested the boosting effect of this chimeric RBD-dimer vaccine in mice after priming with two doses of inactivated vaccines, compared with a booster of inactivated vaccine or ZF2001. The results demonstrated that boosting with bivalent Delta-Omicron BA.1 vaccine greatly promoted the neutralizing activity of the sera to all tested SARS-CoV-2 variants. Therefore, the Delta-Omicron chimeric RBD-dimer vaccine is a feasible booster for those with prior vaccination of COVID-19 inactivated vaccines.

## Dear editor

The severe acute respiratory syndrome coronavirus 2 (SARS-CoV-2) Omicron variant of concern (VOC) is continuously dominating the current pandemic. The sub-variants of Omicron VOC contain far more mutations in spike (S) protein compared with their preceding VOCs (Figure S1) and showed largely reduced sensitivity to the currently-available vaccines [1,2]. In China, more than a billion people have received two or three doses of coronavirus disease 2019 (COVID-19) vaccines, and most of them took inactivated vaccines. We previously developed a protein subunit COVID-19 vaccine ZF2001, using the immunogen of tandem-repeat dimeric receptor-binding domain (RBD), which was approved in China and other countries [3-5]. ZF2001 was also approved as a booster for those who had received two doses of inactivated vaccine (e.g. BBIBP-CorV). Both these inactivated and protein subunit vaccines are based on the early SARS-CoV-2 strain identified in Wuhan (prototype) and elicited sera with greatly reduced neutralization to Omicron [1].

To adapt SARS-CoV-2 variants, we further developed a chimeric Delta-Omicron BA.1 RBD-dimer immunogen which elicited broader sera neutralization of SARS-CoV-2 variants and conferred better protection in mice in comparison to the prototypic homodimer immunogen being used in ZF2001 [6]. COVID-19 protein subunit vaccine ZF2202, based on the chimeric Delta-Omicron BA.1 RBD-dimer antigen, has been approved for clinical trial studies in November 2022 (NCT05616754). Here, we tested the boosting effect of this chimeric RBD-dimer vaccine in mice after receipt of two doses of inactivated vaccines, compared with either the homologous boosting with the inactivated vaccine or the heterologous boosting with ZF2001 (prototype RBD-dimer).

**Figure 1.**
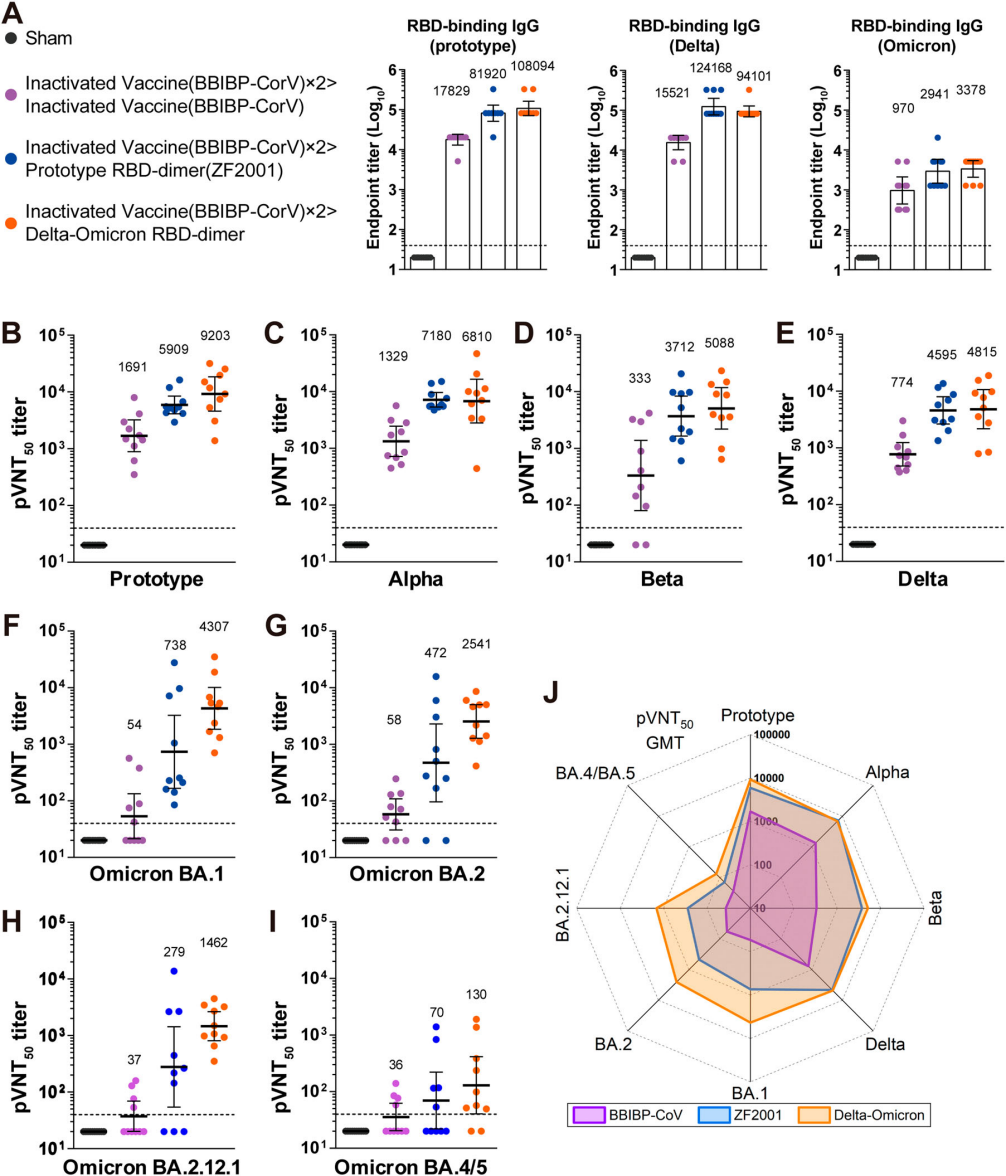
Humoral immune responses after boosting with BBIBP-CorV, ZF2001 or Delta-Omicron BA.1 RBD-dimer in mice. Groups of female BALB/c mice (n = 10) were immunized with two doses of 1·3 U BBIBP-CorV and boosted with homologous 1·3-U BBIBP-CorV, or 5-μg ZF2001, or 5-μg Delta-Omicron BA.1 RBD-dimer (aluminum hydroxide adjuvant) (see schedule shown in Figure S2). Both the BBIBP-CorV and ZF2001 were 1/5 of their dosages used in human. Serum samples were collected according to schedule shown in Figure S2. **(A)** Endpoint titers of antigen-binding IgG after the 3rd jab (day 56). **(B-I)** Sera 50% neutralization titers of pseudotyped viruses displaying spike proteins of prototype **(B)**, Alpha **(C)**, Beta **(D)**, Delta **(E)**, Omicron BA.1 **(F)**, Omicron BA.2 **(G)**, Omicron BA.2.12.1 **(H)** or Omicron BA.4/5 **(I)**. **(J)** Radar plot demonstrating the neutralization profile of sera boosted by BBIBP-CorV, ZF2001 or Delta-Omicron BA.1 RBD-dimer against eight SARS-CoV-2 pseudotyped viruses.

Thirty BALB/c mice were injected with two doses of inactivated vaccine BBIBP-CorV, 21 days apart (Figure S2). Vaccine-elicited RBD-binding antibodies were measured 14 days after dose 2. These 30 mice were evenly distributed into three groups (n = 10) according to the mean titer of RBD-binding IgG for synchronization (Figure S3). Next, each group of mice was given a homologous booster of BBIBP-CorV, a heterologous booster of ZF2001, or a heterologous booster of Delta-Omicron BA.1, at 21 days after the dose two (Figure S2). A group of mice (n = 10) received three doses of sham as control. Serum samples were collected 14 days later to measure the antibody responses. As expected, both homologous and heterologous boosters induced substantial binding IgG to RBD of prototype, Delta, or Omicron. The heterologous boosters were superior to the homologous booster (Figure 1(A)).

Next, we used pseudotyped virus displaying SARS-CoV-2 S protein to evaluate the neutralization activity of vaccine-elicited sera against a panel of strains, including prototype, Alpha (B.1.1.7), Beta (B.1.351), Delta (B.1.617.2) and Omicron (BA.1, BA.2, BA.2.12.1 and BA.4/5 sub-variants). In the BBIBP-CorV booster group, neutralizing seroconversion rate was 100% against pseudovirus displaying prototype, Alpha, and Delta pseudoviruses, reduced to 80% against Beta, and further decreased to 40-70% against four Omicron sub-variants ( Figure 1 (B-I); Table S1). In the ZF2001 booster group, neutralizing seroconversion rate was 100% against five pseudoviruses (prototype, Alpha, Beta, Delta and Omicron BA.1), and reduced to 80%, 70%, and 50% against Omicron sub-variants BA.2, BA.2.12.1, and BA.4/BA.5, respectively. By contrast, the Delta-Omicron BA.1 booster induced 100% seroconversion against all pseudoviruses except Omicron BA.4/5 (seroconversion rate, 80%) ( Figure 1 (B-I); Table S1).

Regarding the sera neutralization titers, in the BBIBP-CorV booster group, the geometric mean titer (GMT) of 50% pseudovirus neutralization was 1691 against the prototype, reduced with 1.3–5.1 folds against early pandemic VOCs (GMT to Alpha, 1329; Beta, 333; Delta, 774), and largely declined with 29.2–47.0 folds against the Omicron sub-variants BA.1, BA.2, BA.2.12.1 and BA.4/5 (GMT from 36-58) ( Figure 1 (B-I) and Figure S4). Comparably, in the ZF2001 booster group, the sera neutralization titers against all SARS-CoV-2 were higher against all preceding VOCs and the Omicron sub-variants ( Figure 1 (B-I)) (GMTs: prototype, 5909; Alpha, 7180; Beta, 3712; Delta, 4595; Omicron BA.1, 738; BA.2, 472; BA.2.12.1, 279; BA.4/BA.5, 70). Impressively, in the Delta-Omicron BA.1 RBD-dimer booster group, the sera neutralization GMTs were largely preserved (if not slightly enhanced) against all early pandemic VOCs in comparison to the ZF2001 booster group, but highly augmented against the Omicron sub-variants (GMTs: BA.1, 4307; BA.2, 2541; BA.2.12.1, 1462; BA.4/5, 130) ( Figure 1 (B-I) and Figure S4). Intuitionally, the radar plot demonstrated that the Delta-Omicron BA.1 RBD-dimer booster induced the broadest and most balanced neutralizing activity against SARS-CoV-2, compared with the homologous booster with the inactivated vaccine or the heterologous booster with prototype RBD-dimer vaccine ( Figure 1 (J)).

We observed that the neutralizing titers against BA.4/5 were lower than the other tested Omicron variants. Compared with BA.1, BA.2 and BA.2.12.1, BA.4/5 contains more mutations in the S protein (Figure S1), resulting in further immune evasion from immunity induced by early-approved COVID-19 vaccines [7-10]. In addition, RBDs of BA.1, BA.2 and BA.4/5 showed different antigenic profiles. It was reported that the vaccine breakthrough infection by BA.1 induced cross-reactive antibodies and new clones of antibodies. They showed decreased neutralizing activities against BA.4/5 due to the extra mutations on RBD, such as D405N, L452R and F486V [7,8,10]. However, the head-to-head comparison showed that chimeric Delta-Omicron BA.1 RBD-dimer induced a broader neutralizing profile than inactivated vaccine and prototype RBD-dimer vaccine (ZF2001) (Figure 1(J)).

Splenic cellular immune responses after sequential vaccination were also measured 14 days after boosting. Peptide pools spanning either the prototype or Omicron antigen elicited substantial secretion of Th1 cytokines (IFN-γ and IL-2) in all booster groups (Figure S5). The levels of cellular immunity against Omicron BA.1 induced by a booster of homologous inactivated vaccine or heterologous Delta-Omicron RBD-dimer vaccine were similar without significant difference (Figure S5). Recently, a similar finding was also reported from the clinical study of BA.5 bivalent mRNA vaccine (Pfizer/BioNTech and Moderna) boosters [11]. In addition, inactivated vaccine and the RBD-dimer protein subunit vaccines are all adjuvanted by aluminum hydroxide. The cellular immune responses elicited by them were not at robust levels, though substantial responses were detected.

Thanks to the rapid vaccine development and mass vaccination campaign worldwide, most people have acquired specific immunity against SARS-CoV-2. However, the breakthrough infections by the Omicron sub-variants are currently the major challenge for pandemic control. Recent studies in macaques indicated that boost vaccination with an Omicron-matched vaccine did not augment sera neutralization against the Omicron variant, compared with boosting with prototype vaccine [12]. Therefore, it is yet unclear what formulations should be used for future COVID-19 vaccines: a booster using the available prototype vaccine, Omicron-matched monovalent vaccine or the multivalent vaccine?

Our results demonstrated that boosting with prototype vaccines had limited augment of sera neutralization of Omicron VOCs, in particular, the sub-variants BA.2.12.1 and BA.4/BA5 in the animal model ( Figure 1 (H, I)). By contrast, boosting with multivalent Delta-Omicron BA.1 vaccine greatly promoted the neutralizing activity of the sera to all tested Omicron sub-variants, yet largely preserved the activity to all early VOCs.

The Omicron-containing bivalent COVID-19 vaccines, developed by Pfizer/BioNTech and Moderna, have been approved for use in the United States and other countries. The published data showed that a booster of bivalent (prototype + Omicron BA.1) mRNA vaccines induced broad antibody responses against SARS-CoV-2 variants, which were superior to prototype sequence-based vaccines (BNT162b2 and mRNA-1273) [13,14]. In addition, another study analyzed the effect of boosting immunization of bivalent protein vaccines (produced by Livzon, China) after three doses of inactivated vaccines and found that bivalent BV-01-B5 vaccine induced stronger neutralizing responses against multiple Omicron sublineages than monovalent V-01 vaccine (prototype) and homologous inactivated vaccine in human participants [15]. These conclusions were similar to ours from the murine model study. However, the bivalent mRNA vaccines from Pfizer/BioNTech and Moderna and protein vaccine BV-01-B5 from Livzon were produced with a 1:1 mixture formulation. The chimeric RBD-heterodimer design strategy allows the single antigen production as the bivalent vaccine. In the context of uncertain future evolutionary steps for SARS-CoV-2 variants, our data support the advice by World Health Organization (WHO) for developing multivalent variant-adapted vaccines to elicit broader immune responses against both co-circulating and emerging SARS-CoV-2 variants [16]. The Delta-Omicron chimeric RBD-dimer vaccine is a feasible booster for those with prior vaccination of COVID-19 inactivated vaccines.

Given that the sequences of influenza vaccines were updated annually with the data from global surveillance and prediction, the strategy of future updated COVID-19 vaccines will possibly be similar to those from influenza vaccines. Currently, the Omicron sub-variants are co-circulating in the world, including XBB, BQ.1.1 and BF.7 with strong immune evasion. Next-generation and updated vaccines were urgently needed. Our study provided a bivalent booster vaccine candidate with high and broad-spectrum immunogenicity.

## Supplementary Material

Supplemental Material
