# Supplementary material for: A booster of Delta-Omicron RBD-dimer protein subunit vaccine augments sera neutralization of Omicron sub-variants BA.1/BA.2/BA.2.12.1/BA.4/BA.5
Source: Emerg Microbes Infect. 2023 Feb 27;12(1):e2179357. doi: 10.1080/22221751.2023.2179357 (PMC9979983; doi:10.1080/22221751.2023.2179357)

**Supplementary materials**

**A booster of Delta-Omicron RBD-dimer protein subunit vaccine augments sera neutralization of Omicron sub-variants BA.1/BA.2/BA.2.12.1/BA.4/BA.5**

**Author and affiliations**

Minrun Duan^a^, Huixin Duan^b^, Yaling An^b^, Tianyi Zheng^c^, Shengfeng Wan^d^, Hui Wang^e^, Xin Zhao^d^, Lianpan Dai^b,d^*, Kun Xu^f^*, George F. Gao^b,d,f^

^a^School of Life Sciences, Yunnan University, Kunming 650091, China

^b^Savaid Medical School, University of Chinese Academy of Sciences, Beijing 101408, China

^c^Zhejiang University School of Medicine, Hangzhou 310058, China

^d^CAS Key Laboratory of Pathogen Microbiology and Immunology, Institute of Microbiology, Chinese Academy of Sciences, Beijing 100101, China

^e^Beijing Institute of Biological Products Company Limited, Beijing 100176, China

^f^Research Network of Immunity and Health (RNIH), Beijing Institutes of Life Science, Chinese Academy of Sciences, Beijing 100101, China

*Corresponding author. Email: dailp@im.ac.cn (L.D.); xukun@biols.ac.cn (K.X.)

**Materials and Methods**

**Vaccines**

Inactivated virus vaccine BBIBP-CoV was produced by Beijing Institute of Biological Products, Sinopharm [1,2]. Protein subunit vaccine ZF2001 was produced by Anhui Zhifei Longcom Biopharmaceutical Co., Ltd [3,4]. SARS-CoV-2 Delta-Omicron chimeric RBD-dimer was one Delta RBD (S protein residues 319–537, GenBank: OK091006.1) and one Omicron BA.1 RBD (S protein residues 316–534, GISAID: EPI_ISL_6795848) connected as tandem repeat. The Delta-Omicron chimeric RBD-dimer protein was produced in our lab as previously described [5] and adjuvanted by aluminum hydroxide.

**Animals**

Specific pathogen-free (SPF) female BALB/c mice (7-8 weeks old) were purchased from Beijing Vital River Laboratory Animal Technology Co., Ltd. (licensed by Charles River) and housed under SPF conditions in the laboratory animal facilities at Institute of Microbiology, Chinese Academy of Sciences (IMCAS). All mice were allowed free access to water and standard chow diet and provided with a 12-hour light and dark cycle. The mice experiments were approved by the Committee on the Ethics of Animal Experiments of the IMCAS, and performed in compliance with the recommendations in the Guide for the Care and Use of Laboratory Animals of the IMCAS Ethics Committee.

The mice were immunized intramuscularly three jabs with 21 days apart. Blood samples were collected at 14 days post the second jab and 14 days post the third jab.

**ELISA**

Binding properties of sera to SARS-CoV-2 RBD protein were determined by ELISA as previously described [5], with some modifications. Briefly, 96-well plates were coated over-night with 3 μg/ml of SARS-CoV-2 prototype, Delta, or Omicron (BA.1) RBD protein in 0.05 M carbonate-bicarbonate buffer (pH 9.6) and blocked in 5% skim milk in PBS. Serum samples from mice were serially diluted and added to each well. The plates were incubated for 2 hours and then washed. The plates were incubated with goat anti-mouse IgG-HRP antibody for 1.5 hours and then washed. The plates subsequently developed with 3,3’,5,5’-tetramethylbenzidine (TMB) substrate. Reactions were stopped with 2 M hydrochloric acid, and the absorbance was measured at 450 nm using a microplate reader (PerkinElmer, USA). The endpoint titers were defined as the highest reciprocal dilution of serum to give an absorbance greater than 2.5-fold of the background values. Antibody titer below the limit of detection was determined as half the limit of detection.

**Pseudotyped virus neutralization assay**

Vero-E6 cells were maintained in Dulbecco’s modified Eagle’s medium (DMEM, Invitrogen, USA) supplemented with 10% fetal bovine serum (FBS) at 37°C under 5% CO_2_. The pseudotyped viruses displaying SARS-CoV-2 spikes express GFP in infected cells. The methods for preparing pseudotyped viruses and neutralization assays were described previously [6]. Mice sera were 2-fold serially diluted and incubated with pseudotyped virus at 37°C for 1 hour. Then the mixture was transferred to pre-plated Vero-E6 cell monolayers in 96-well plates. After incubation for 15 hours, the transducing unit numbers were calculated on a CQ1 confocal image cytometer (Yokogawa). Fifty percent pseudovirus neutralization titer (pVNT_50_) was determined by fitting nonlinear regression curves using GraphPad Prism and calculating the reciprocal of the serum dilution required for 50% neutralization of infection. pVNT_50_ below the limit of detection was determined as half the limit of detection.

**ELISpot assays**

IFNγ-, IL-2-, and IL-4-based ELISpot assays were performed using the mouse IFNγ, IL-2 and IL-4 ELISpot kits (Mabtech) following the manufacturer’s instructions. Briefly, mice spleens were collected at 14 days after the third vaccination. MultiScreen HTS IP Filter Plates (Millipore Sigma #MSIPS4W10) were precoated with anti-mouse IFNγ, IL-2, or IL-4 antibody overnight at 4°C. The plates were then washed twice and blocked for 1 hour with RPMI 1640 medium at room temperature. Mouse splenocytes were plated and mixed with the peptide pool (2 μg/mL) consisting of 15–18-mers (overlapping by 11 amino acids) and spanning S protein of prototype SARS-CoV-2 or the RBD of Omicron variant. After incubation for 40 hours at 37°C, the cells were removed, and the plates were processed in turn with biotinylated detection antibody, streptavidin-ALP conjugate, and substrate. The numbers of the spots were determined using an automatic ELISpot reader and image analysis software (Cellular Technology Ltd.).

**References:**

1. Wang H, Zhang Y, Huang B, et al. Development of an inactivated vaccine candidate, BBIBP-CorV, with potent protection against SARS-CoV-2. Cell 2020; 182(3): 713-721.e9.

2. Al Kaabi N, Zhang Y, Xia S, et al. Effect of 2 inactivated SARS-CoV-2 vaccines on symptomatic COVID-19 infection in adults: a randomized clinical trial. JAMA 2021; 326(1):35-45.

3. Dai L, Zheng T, Xu K, et al. A universal design of betacoronavirus vaccines against COVID-19, MERS, and SARS. Cell 2020; 182(3):722-733.e11.

4. Dai L, Gao L, Tao L et al. Efficacy and safety of the RBD-dimer-based Covid-19 vaccine ZF2001 in adults. N Engl J Med 2022; 386(22):2097-2111.

5. Xu K, Gao P, Liu S et al. Protective prototype-Beta and Delta-Omicron chimeric RBD-dimer vaccines against SARS-CoV-2. Cell 2022; 185(13):2265-2278.e14.

6. Zhao X, Li D, Ruan W et al. Effects of a prolonged booster interval on neutralization of Omicron variant. N Engl J Med 2022; 386(9):894-896.


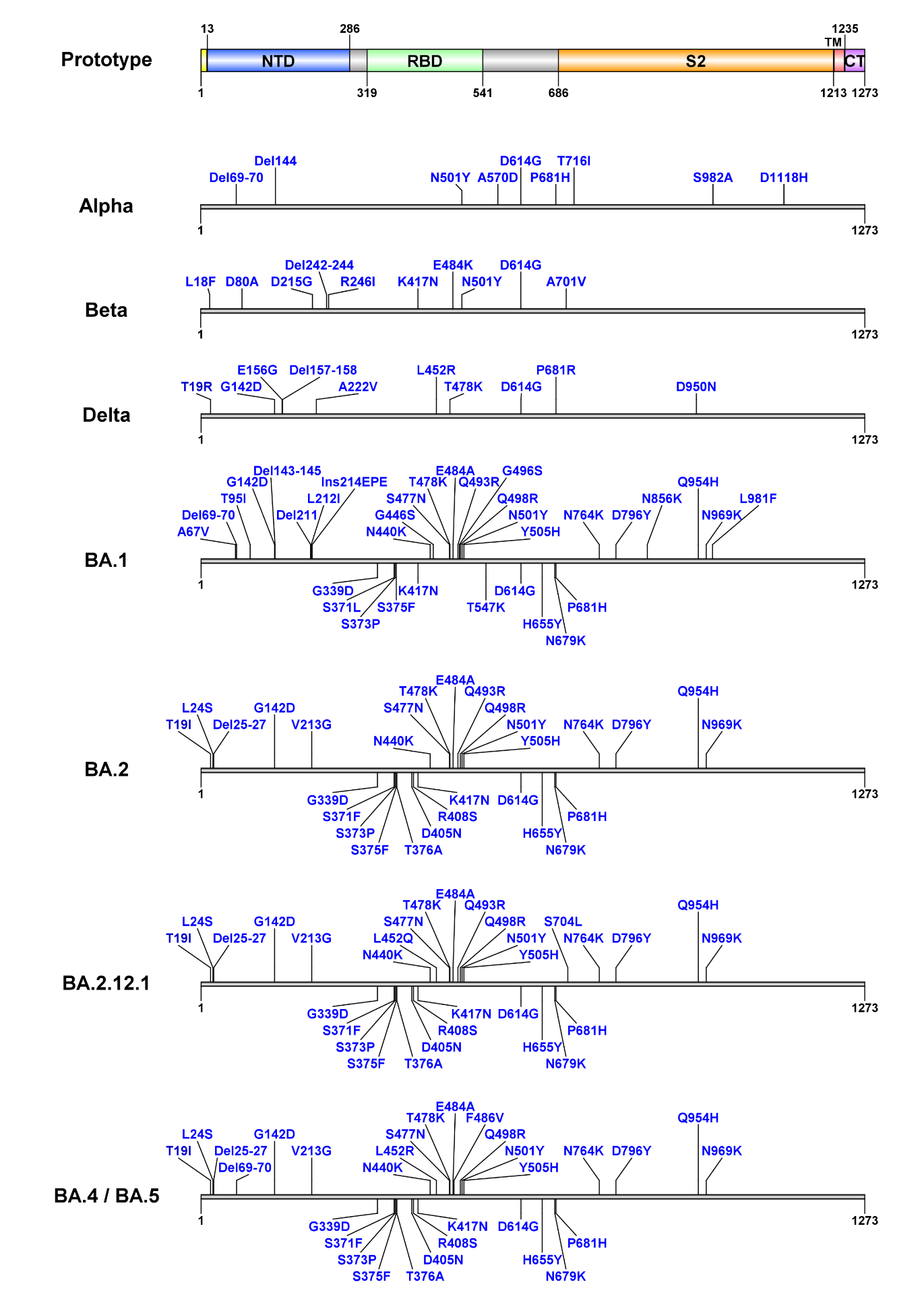


**Figure S1**: Schematic representation of SARS-CoV-2 spike proteins used in this study.

The prototype sequence is from IVDC-HB-01/2019 strain (GISAID: EPI_ISL_402119). The mutation sites of the variants were indicated. NTD, N-terminal domain; RBD, receptor-binding domain; TM, transmembrane domain; CT, cytoplasmic tail.


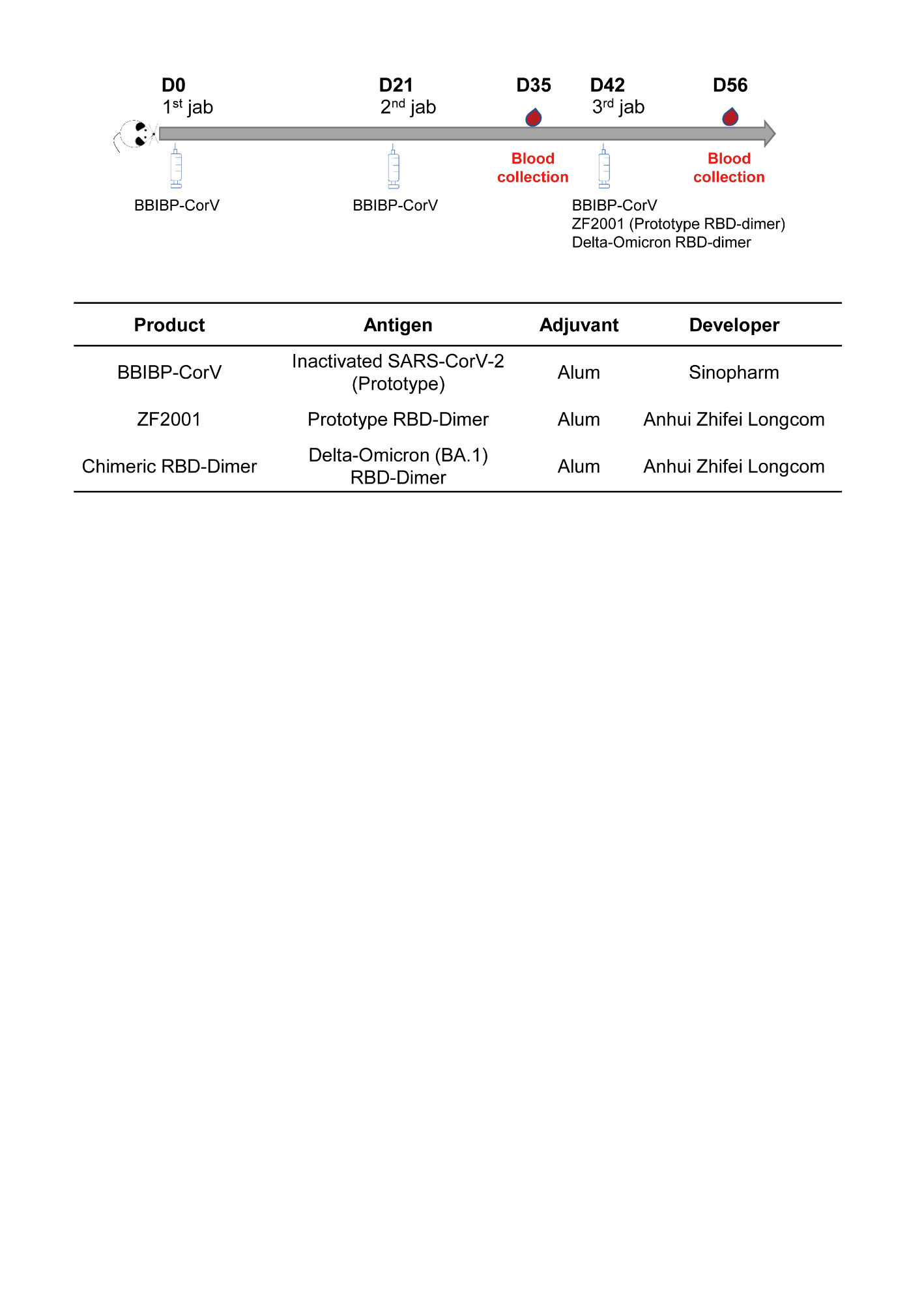


**Figure S2**: Time courses of vaccine immunization and sampling and details of the vaccines.

Four groups (n = 10) of female BALB/c mice were injected intramuscularly with 21 days apart. Serum samples were collected at 14 days post the second jab and 14 days post the third jab. The antigens, adjuvants and developers of the vaccines used in this study were listed in the table.


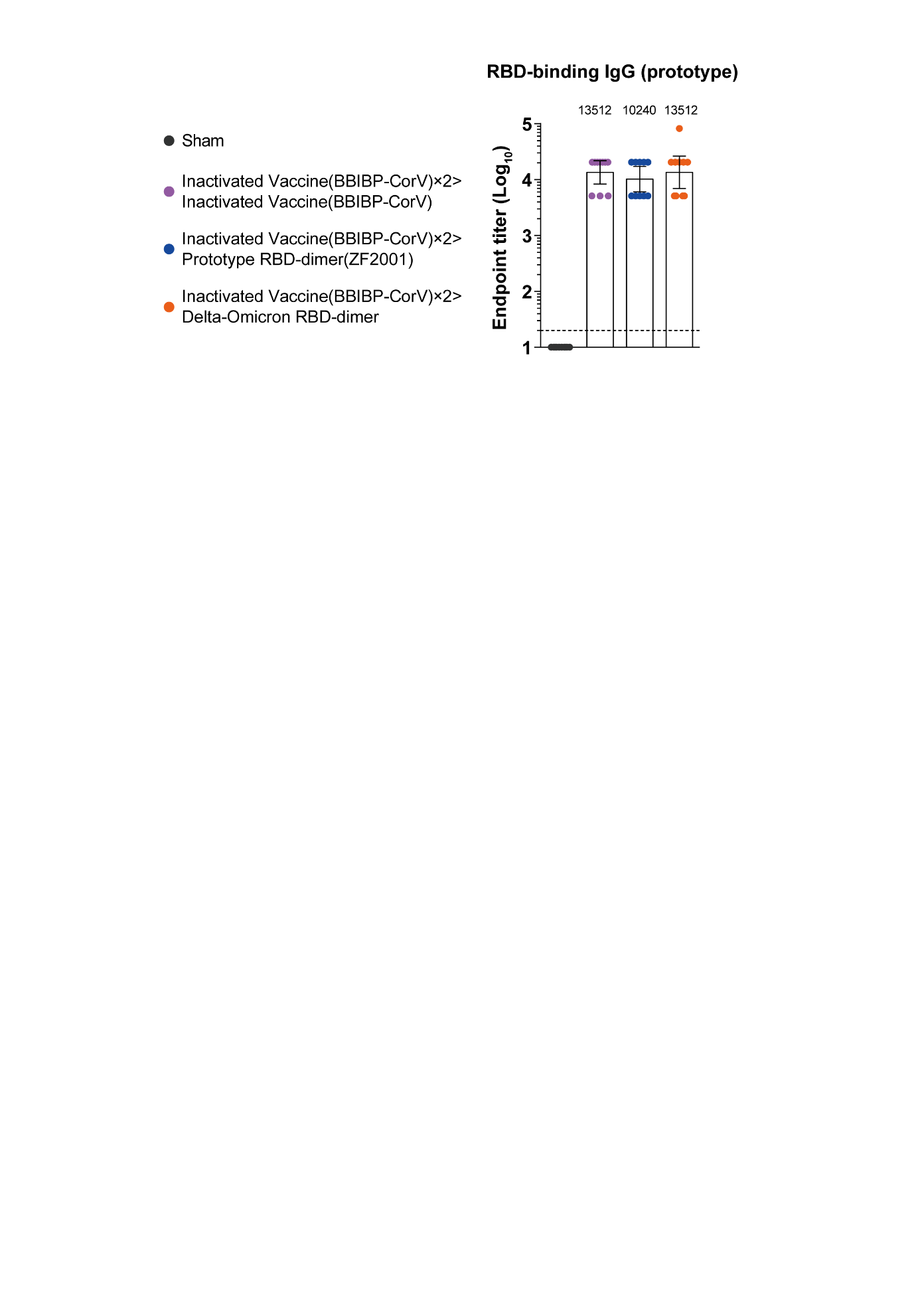


**Figure S3**: Serological RBD-binding IgG titers after the second jab vaccination.

Measurement of prototype SARS-CoV-2 RBD-binding IgG endpoint titers of serum samples collected at 14 days after the second jab vaccination. The values are the GMT ± 95% confidence interval (CI). The horizontal dashed line indicates the limit of detection.


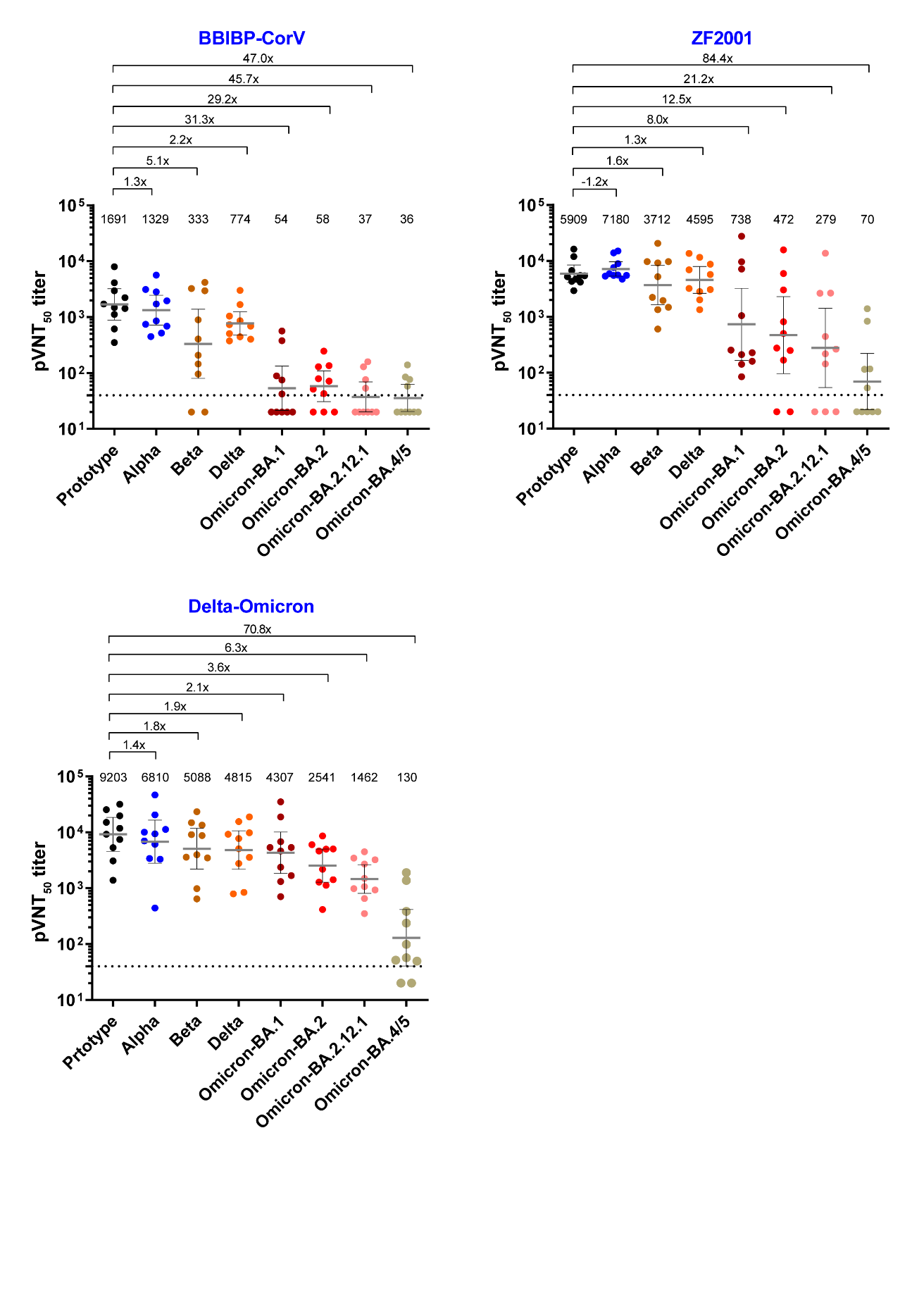


**Figure S4**: Pseudovirus neutralization titers.

The pVNT_50_ of sera collected at 14 days after the third jab vaccination against SARS-CoV-2 prototype and variants pseudoviruses were measured and analyzed. The variants include Alpha, Beta, Delta, Omicron BA.1, BA.2, BA.2.12.1 and BA.4/5. The values are the GMT ± 95% CI. The folds were calculated as the GMTs against prototype: GMTs against variants, and the minus sign indicated that the value of GMT against variant was greater than that against prototype. The horizontal dashed line indicates the limit of detection.


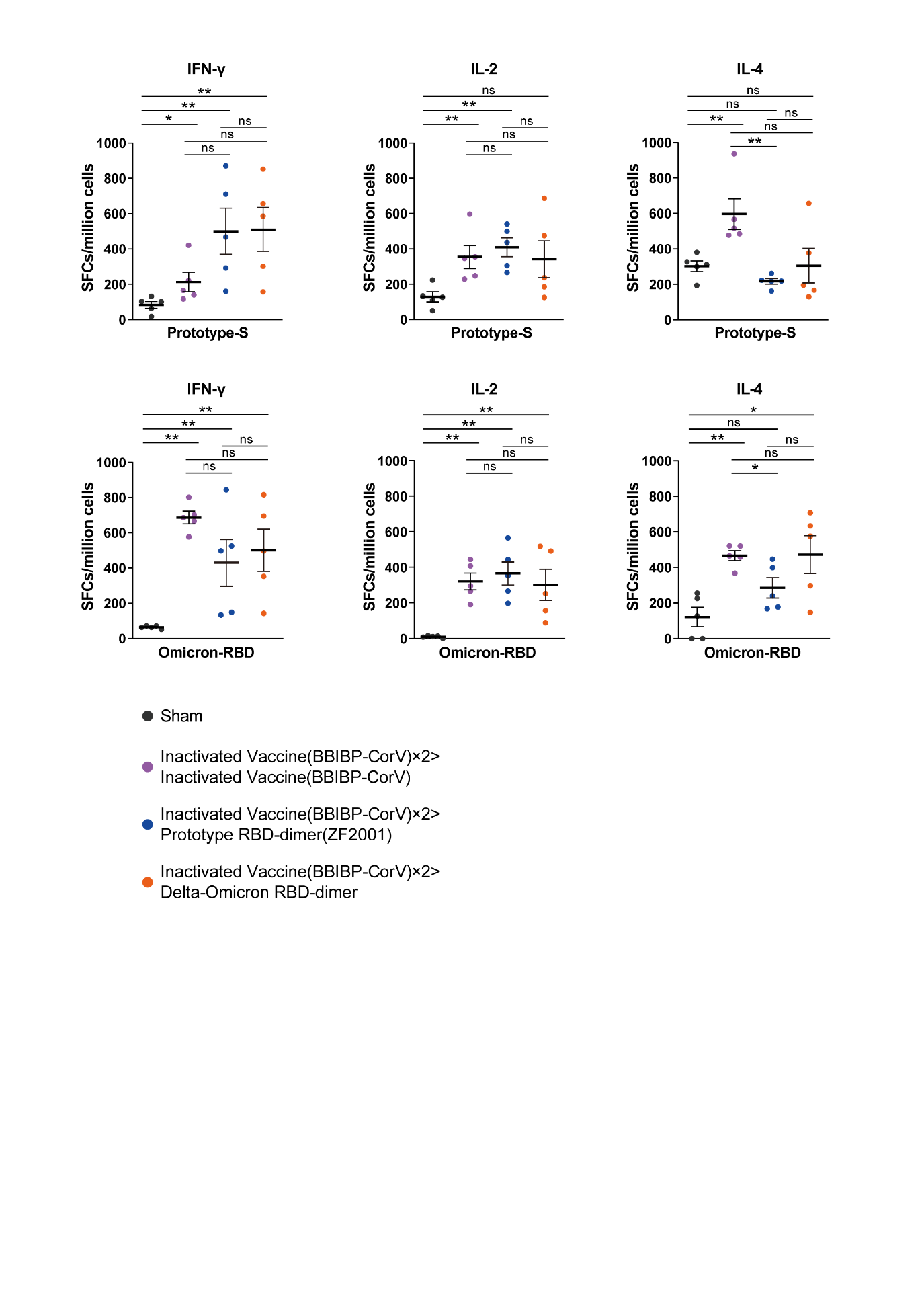


**Figure S5**: Cellular immune responses.

Measurement of the IFNγ, IL-2, and IL-4 secretion of mouse splenocytes after stimulation with SARS-CoV-2 prototype S peptide pool or Omicron BA.1 RBD peptide pool by ELISpot assays. *P*-values were analyzed with two-tailed Mann Whitney test (ns, *p* > 0.05; **p* < 0.05; ***p* < 0.01).

**Table S1**. The serological pVNT_50_ GMT titers and proportions of samples with positive neutralization.


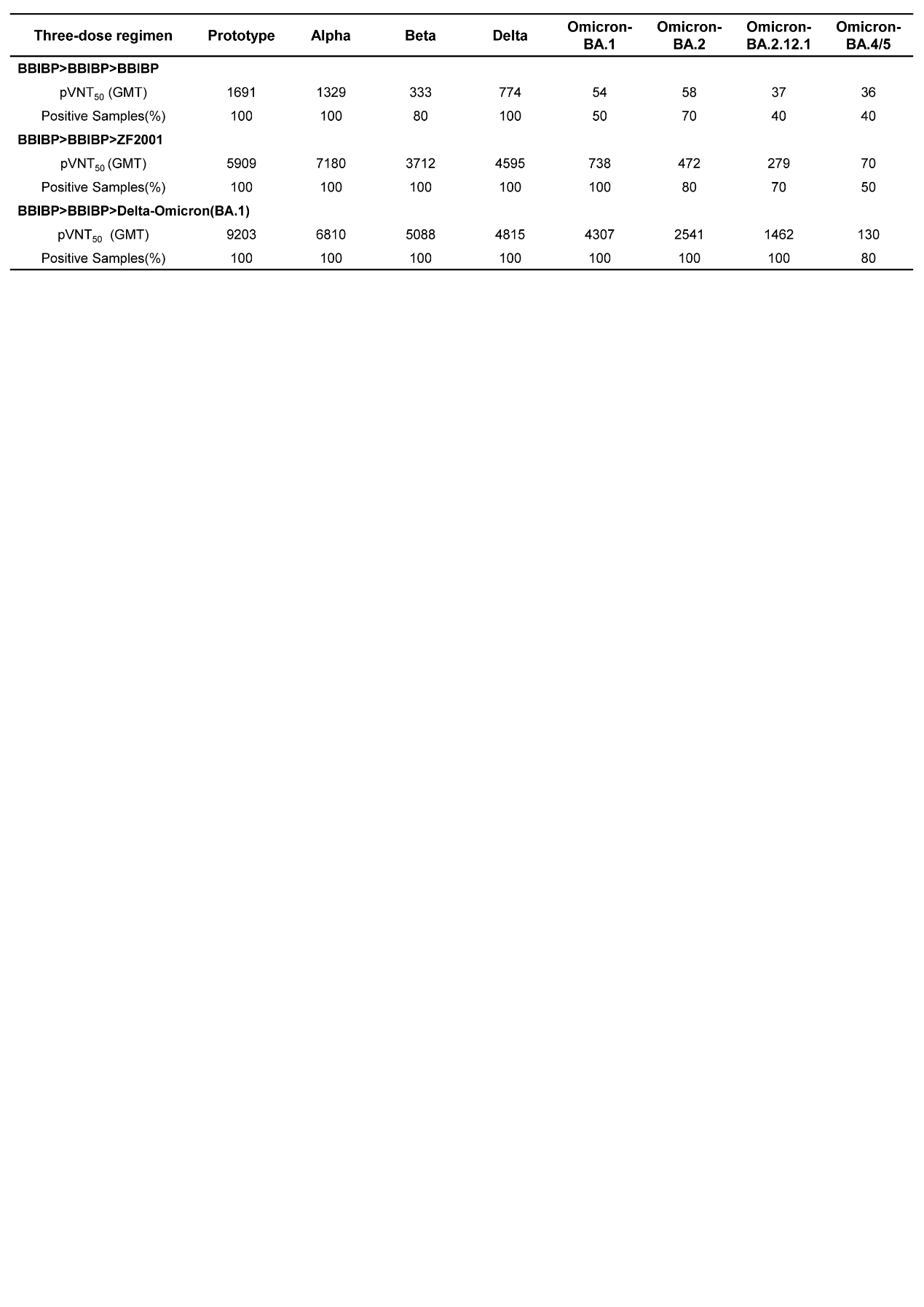

Supplement: Supplemental Material [file TEMI_A_2179357_SM8985.docx]
